# Effect of variety and drying method on the nutritive value of corn for growing pigs

**DOI:** 10.1186/2049-1891-5-18

**Published:** 2014-03-22

**Authors:** Quanfeng Li, Meng Shi, Chuanxin Shi, Dewen Liu, Xiangshu Piao, Defa Li, Changhua Lai

**Affiliations:** 1 State Key Laboratory of Animal Nutrition, Ministry of Agriculture Feed Industry Centre, China Agricultural University, Beijing 100193, China

**Keywords:** Corn, Digestible energy, Drying method, Pigs, Variety

## Abstract

**Background:**

This experiment was conducted to determine the nutritive value of corn from the north of China for growing pigs. The experiment examined corn variety (LS1, LS2, LS3 and LS4) grown in one location, drying method (sun dried and artificially dried) and different drying temperatures. Corn harvested at 20-25% moisture was dried to about 12% moisture by sun drying and artificially drying at 80, 100, or 120°C in a fluidized bed dryer. Ninety-six barrows (average BW of 33.4 ± 2.7 kg) were housed in individual metabolism crates to facilitate separate collection of feces and urine. A five-day collection period followed a seven-day diet acclimation period.

**Results:**

The results indicated that variety significantly influenced (*P* < 0.01) the 1,000 kernel weight of corn but not the bulk weight. Variety also influenced the available energy content (digestible energy of dry matter, *P* < 0.01; metabolisable energy of dry matter, *P* < 0.01) and digestibility of organic matter (*P* < 0.01), as well as dry matter (*P* < 0.01) and gross energy (GE) content (*P* < 0.02). The drying method of corn significantly influenced the 1,000 kernel weight (*P* < 0.01), bulk weight (*P* < 0.01) and digestibility of ether extract (EE) (*P* < 0.01). No effect of drying temperature on the digestibility of organic matter, dry matter (DM), crude protein (CP), neutral detergent fiber (NDF), acid detergent fiber (ADF) and gross energy was observed, but gelatinization (*P* < 0.05) and test weight (*P* < 0.01) decreased with an increase in temperature.

**Conclusions:**

Variety has a significant impact on the nutritive value of corn for growing pigs, and greater attention needs to be paid to these influences in the assignment of the nutritive value of corn given to growing pigs.

## Background

Corn growers are usually confronted with difficulty in the safe storage of their grain crop because of a high moisture content (22-30%) at the time of harvest. This condition is especially pronounced in the north of China where the weather conditions are unfavorable for natural field drying. So, the purpose of drying is to lower the moisture content in grain to the safe storage content of about 14%. Therefore, most of the corn is artificially dried at a temperature of 120°C in Jilin, Liaoning, and Heilongjiang province, which are the biggest corn producing areas in China. In order to decrease the damage to corn from a high temperature, the low temperature drying process, which occurs at 80°C is used. However, corn in the Shandong and Henan provinces, which are in the east of China, was dried in the sun.

As expected, there were many studies on corn variety in feeds, but the varieties studied were high-oil [1,2], high-lysine [3], low-phytate [4] and so on. There varieties are very special, and the planting area is not large. The varieties of corn used in feeds still are conventional varieties. However, there is a lack of congruent information on the variation in nutritive value as affected by conventional variety, drying method and drying temperature of corn. Therefore, the objectives of this experiment were to measure the influence of conventional variety on the nutritive value of corn and to compare estimates of the effects of drying method on the nutritive value of corn. An additional objective was to evaluate varying temperature during drying on the nutritive value of corn in growing pigs.

## Materials and methods

The parts of this experiment that involved animals were conducted in the Metabolism Laboratory of the Ministry of Agriculture Feed Industry Centre (China Agricultural University, Beijing, China). The protocol for the experiment was reviewed and approved by the Institutional Animal Care and Use Committee at China Agricultural University.

### Selection and preparation of the corn samples

Four corn variety samples (LS1, LS2, LS3 and LS4) were obtained from one location in the north of Hebei province. The four varieties were very widespread, and the planting area was very large. The varieties of hardness were classified as soft (LS1), hard (LS3), and of intermediate hardness (LS2; LS4). Corn was hand-harvested at 20-25% moisture and was spread on a tarp on the ground for a maximum of one week prior to threshing and drying.

Grain samples from each variety were divided into four subplots of 200 kg each. One of the subplots was placed on an airfield and allowed to dry to about 12% moisture content after threshing. The remaining three subplots were dried in a fluidized bed dryer at 80, 100 or 120°C. Final moisture contents were about 12% (ranging from9.4 to 14.7%). At each drying temperature, the varieties were dried in a random order. Approximate drying times were 60, 50 and 40 min, respectively, for temperatures of 80, 100 and 120°C. When samples were removed from the dryer, they were cooled at room temperature before packaging in bags.

### Animals, housing, and experimental design

Ninety-six barrows (initial body weight of 33.4 ± 2.7 kg) were used in this experiment. The pigs were Duroc × Landrace × Yorkshire crossbreeds. Pigs were individually housed in stainless steel metabolism cages (1.4 m × 0.45 m × 0.6 m). A feeder and a nipple drinker were installed in each pen. The crates were located in an environmentally controlled room with a temperature of 22 ± 1°C. Pigs were allotted to one of sixteen diets according to a completely randomized design, and each diet was measured with six pigs.

### Diets, feeding, and sample collection

Sixteen diets were formulated to contain 96.8% of one of each of the corn samples and 3.2% minerals and vitamins (Table 1). Corn was assumed to be the only source of energy in the diet, as the slight contribution of energy from the vitamin and mineral premix was assumed to be negligible. Vitamins, salt, and minerals were included in all diets to meet or exceed the estimated requirements for growing pigs [5].

**Table 1:** Composition of the experimental diets (as-fed basis) fed to growing pigs for comparison of the energy digestibility between different corn samples

**Ingredient, %**	**Experimental diet**
Corn	96.8
Antioxidant^1^	0.1
Calcium phosphate	1.7
Limestone	0.6
Salt	0.3
Vitamin and mineral premix^2^	0.5

^1^Santoquin MAX composite antioxidant, contained no less than 10% Ethoxyquin, no less than 3% ButylatedHydroxytoluene (BHT) and Citric acid, provided by Novus International, Inc.

^2^Premix provided the following per kg of complete diet for growing pigs: vitamin A, 5,512 IU; vitamin D_3_, 2,200 IU; vitamin E, 30 IU; vitamin K_3_, 2.2 mg; vitamin B_12_, 27.6 μg; riboflavin, 4 mg; pantothenic acid, 14 mg; niacin, 30 mg; choline chloride, 400 mg; folacin, 0.7 mg; thiamine 1.5 mg; pyridoxine 3 mg; biotin, 44 μg; Mn, 40 mg (MnO); Fe, 75 mg (FeSO_4_ · H_2_O); Zn, 75 mg (ZnO); Cu, 100 mg (CuSO_4_ · 5H_2_O); I, 0.3 mg (KI); Se, 0.3 mg (Na_2_SeO_3_).

Feed was provided twice daily as a mash, at 0800 and 1700 h. Water was freely available for each pig. During the period of adjustment to the metabolism crates and diets, average daily feed intake was gradually increased until it was estimated to supply 4% of the BW determined at the initiation of each adaptation period. During the collection period, all fresh fecal samples were collected as often as possible throughout the day from all pigs and stored at -20°C. The collection and sample preparation of feces and urine were conducted according to the methods described by Song et al. [2].

### Chemical analyses

At the conclusion of the experiment, the fecal samples were dried at 60°C in a forced-air oven for 72 h. After drying, all samples were finely ground, and ground samples of diets and feces were analyzed for DM [6], ether extract (EE) [7], and ash [6]. Kjeldahl N was determined according to the method of Thiex et al. [8]. The diets and feces samples were analyzed for NDF and ADF, and the content of NDF and ADF were determined using filter bags and fiber analyzer equipment (Fiber Analyzer, Ankom Technology, Macedon, NY) following a modification of the procedure of Van Soest et al. [9]. The gelatinized starch of corn samples was determined by enzymatic hydrolysis as described by Xiong et al. [10]. Urine samples (4 mL) were injected into 2 filter papers in a special crucible and then dried for 8 h in a 65°C drying oven prior to determination of the energy content. The gross energy (GE) of urine was measured by injecting 4 mL of sample into 2 filter papers in a special crucible and then dried for 8 h in a 65°C drying oven to determine the energy content. The GE of feces and diets were measured using an automatic adiabatic oxygen bomb calorimeter (Parr 6400 Calorimeter, Moline, IL).The 1,000 kernel weight (g/1,000 seeds) was measured in each sample of test corn by first cleaning it of all foreign materials and then counting 1,000 seeds. The particle size were determined according to the method ASAE S319.4 (2008) [11].

### Calculations and statistical analyses

The digestible energy (DE), metabolisable energy (ME) and apparent total tract digestibility (ATTD) of nutrients in the 16 feed samples were measured and then later converted to reflect the digestibility of the individual corn samples. The small portion of the experimental diets that consisted of minerals and vitamins (3.2%) was assumed to have a negligible contribution to the digestibility of GE.

The DE and ME contents of the 16 diets were calculated using Eq:DE = (GEi - GEf)/Ft, ME = (GEi – GEf – GEu)/Ft, where DE is the DE content in diets (kcal/kg of DM), GEi is the total GE intake (kcal of DM), GEf is the GE content in feces (kcal of DM), Ft is the total feed intake (kg of DM), ME is the ME content in diets (kcal/kg of DM), and GEu is the GE content in urine (kcal of DM). The apparent total tract digestibility (ATTD, %) for energy, organic matter (OM), DM, EE, ADF, NDF and CP was calculated using Eq: ATTD = [(Fi – Ff)/Fi] × 100, where Fi is the total intake (kcal or g) of respective component in the collection period, and Ff is the total fecal output (kcal or g) of respective component originating from the feed that was given during the collection period. Data were analyzed as a randomized complete block design by using the PROC MIXED procedure (SAS Inst. Inc., Cary, NC) with pig as the experimental unit. Main effects of variety and drying method interactions were tested. Orthogonal polynomial contrasts were used to detect linear and quadratic responses to drying temperature. Means were separated using the LSMeans procedure and the PDIFF option of SAS. Pig was the experimental unit for all calculations, and an alpha level of 0.05 was used to assess the significance of difference between means.

## Results

The mean physical characterization and chemical composition of the four corn varieties are presented in Table 2. The bulk weight of corn ranged from 716.1 to 765.8 g/L, with a mean value of 744.6 g/L (CV 3.0%). The 1,000 kernel weight was highly variable, and the content ranged from 282.2 to 356.6 g with a mean value of 318.4 g (CV 10.1%).

**Table 2:** **Physical and chemical characterization of the corn samples**^**a**^

**Variety**	**Drying method**	**1,000 kernel weight, g**	**Bulk weight g/L**	**DM, %**	**EE, %**	**NDF, %**	**ADF, %**	**CP, %**	**Ash, %**	**GE, MJ/kg**	**Starch gelatinization, %**	**Particle size, μm**
LS1	Sundried	330.4	716.2	85.35	3.17	9.15	2.11	7.79	1.25	15.88	12.34	538.09 ± 1.11
	80°C	330.7	699.5	88.00	3.52	9.05	1.86	7.84	1.21	16.27	17.26	549.78 ± 1.12
	100°C	317.0	662.0	90.63	3.51	9.03	2.20	7.80	1.35	16.92	18.60	619.47 ± 1.11
	120°C	313.3	682.3	90.24	3.43	8.82	1.98	7.73	1.22	16.81	12.20	578.91 ± 1.12
LS2	Sundried	282.2	758.6	85.14	3.15	10.28	2.24	7.34	1.12	15.83	14.50	520.46 ± 1.10
	80°C	269.5	716.9	88.01	3.74	10.03	2.12	7.38	1.20	16.45	19.92	501.04 ± 1.12
	100°C	279.5	692.3	88.21	3.57	9.98	1.90	7.81	1.12	16.31	15.61	481.23 ± 1.09
	120°C	273.9	670.5	89.77	3.78	10.05	2.16	7.28	1.19	16.60	13.46	506.32 ± 1.11
LS3	Sundried	304.6	738.2	85.93	3.21	10.49	2.27	7.11	1.17	16.00	15.62	541.38 ± 1.12
	80°C	294.8	701.9	88.45	3.18	10.27	2.25	7.50	1.11	16.50	15.82	525.26 ± 1.12
	100°C	296.3	678.8	88.93	3.50	10.65	2.37	7.25	1.20	16.45	19.67	512.40 ± 1.12
	120°C	286.2	664.5	89.33	3.49	10.04	2.17	7.70	1.28	16.57	13.19	379.59 ± 1.14
LS4	Sundried	356.6	765.9	86.30	3.04	9.85	2.13	8.60	1.11	15.96	13.82	518.24 ± 1.11
	80°C	317.4	692.8	88.53	2.85	9.42	2.15	8.91	1.17	16.37	13.73	511.25 ± 1.13
	100°C	309.7	681.5	89.10	2.81	10.16	2.57	8.32	1.27	16.48	13.54	496.71 ± 1.12
	120°C	316.5	655.5	89.50	2.68	10.28	2.14	8.94	1.29	16.55	12.57	413.96 ± 1.10

^a^All data are the results of a chemical analysis conducted in duplicate.

Drying the corn samples using different temperatures affected certain characteristics of the grain kernels. In a blind taste-comparison test, five individuals were given these samples of corn to taste. Test subjects found that a progressive brown darkening and parched corn odor and taste occurred in corn dried at 120°C. Even though the same hammer mill and screen were used in all cases in the experiment, the particle size of corn decreased as drying temperatures increased, except for the variety of LS1. The EE, NDF, ADF, CP and ash contents were not significantly affected by drying temperature (Table 2). However, the DM content of corn increased linearly as drying temperature increased (linear, *P* < 0.01; quadratic, *P* < 0.03).The difference between two drying methods was significant with regard to bulk weight and 1,000 kernel weight (*P* < 0.01). However, increasing the drying temperature decreased the bulk weight (linear, *P* < 0.01; quadratic, *P* < 0.01). There was no difference between varieties and drying methods for starch gelatinization (Table 3).

**Table 3:** Effect of variety and drying method on the physical characteristics and starch gelatinization of corns for growing pigs

**Item**	**Variety**				**Sun dried**	**Artificially dried °C**			**SEM**	***P*-value**^**1**^			
										**Variety**	**Drying method**^**2**^	**Air temperature**	
	**LS1**	**LS2**	**LS3**	**LS4**		**80**	**100**	**120**				**Linear**	**Quadratic**
Bulk weight, g	690.0	695.8	709.5	698.92	744.6	702.7	678.6	668.1	10.37	0.58	0.01	0.01	0.01
1,000 kernel weight, g/kg	322.8^a^	295.4^b^	276.2^c^	325.0^a^	318.4	303.1	300.6	297.4	4.70	0.01	0.01	0.71	0.94
Starch gelatinization of DM, %	17.03	18.24	18.09	15.19	16.42	18.91	18.88	14.33	1.35	0.48	0.37	0.06	0.06
DM, %	88.36	87.78	88.16	88.56	85.68	88.25	89.22	89.71	0.84	0.62	0.01	0.01	0.03
GE, MJ/kg	16.34	16.30	16.38	16.47	15.92	16.40	16.54	16.63	0.18	0.57	0.01	0.08	0.22
Particle size, μm	485.04	502.26	489.66	571.56	529.54	521.83	527.45	469.70	49.24	0.10	0.43	0.27	0.42

^1^Interaction between variety and drying method was not significant (*P* > 0.10).

^2^Contrast for Artificial dried vs. sun dried.

^a-c^Means followed by the same letter within each row are not significantly different from each other (*P* > 0.05).

The DE and ME content of the 16 test corn samples and digestibility of nutrients are presented in Table 4. The interaction term between variety and drying method was not significant. The LS1 variety had a higher digestible energy than LS2 (*P* < 0.05); while no differences among LS2, LS3 and LS4 were observed. The ATTD for OM, DM and GE in LS1 were greater (*P* < 0.05) than in all other corn varieties while no differences among LS2, LS3 and LS4 were observed. Acid detergent fiber and NDF digestibility coefficients varied from 34.6 to 51.3% and 45.2 to 51.0%, respectively, for the four varieties. The EE and CP fractions were digested to a much greater degree than the structural carbohydrates. Drying method did not affect the digestible energy content and digestibility of nutrients, with the exception of DE (as fed basis), ME (as fed basis) and the ATTD of EE.

**Table 4:** Effect of variety and drying method on the nutritive value of corns for growing pigs

**Item**	**Variety**				**Sun dried**	**Artificially dried °C**			**SEM**	***P*-value**^**1**^			
										**Variety**	**Drying method**^**2**^	**Air temperature**	
	**LS1**	**LS2**	**LS3**	**LS4**		**80**	**100**	**120**				**Linear**	**Quadratic**
DE of DM, MJ/kg	16.14^a^	15.86^b^	15.86^b^	15.82^b^	15.82	16.03	15.92	15.91	0.06	0.01	0.06	0.23	0.45
ME of DM, MJ/kg	15.87^a^	15.51^b^	15.61^b^	15.51^b^	15.57	15.76	15.63	15.61	0.06	0.01	0.06	0.19	0.40
Digestibility coefficients, %													
Organic matter	91.56^a^	90.10^b^	90.21^b^	90.03^b^	90.24	90.77	90.48	90.41	0.27	0.01	0.31	0.45	0.73
Dry matter	90.03^a^	88.54^b^	88.70^b^	88.62^b^	88.66	89.43	88.96	88.85	0.30	0.01	0.23	0.20	0.41
Energy	89.58^a^	88.11^b^	88.00^b^	88.24^b^	88.02	89.04	88.47	88.41	0.34	0.02	0.13	0.21	0.40
NDF	57.01	48.20	45.78	45.24	52.28	50.20	46.33	47.43	3.35	0.09	0.27	0.62	0.78
ADF	51.32	41.94	34.65	34.58	48.54	40.92	36.27	36.76	4.47	0.06	0.06	0.57	0.79
Crude protein	80.81	78.56	76.76	78.56	78.99	79.37	77.17	79.16	1.18	0.15	0.75	0.91	0.38
Ether extract	61.13	65.25	64.07	63.71	59.18	66.46	66.83	67.68	1.39	0.31	0.01	0.53	0.82

^1^Interaction between variety and drying method was not significant (*P* > 0.10).

^2^Contrast for Artificial dried vs. sun dried.

^a-b^Means followed by the same letter within each row are not significantly different from each other (*P* > 0.05).

In the 16 corn samples, fibrous compounds had a negative correlation with DE and ME content, while the correlation of GE with DE and ME content was positive (Table 5). The NDF content had the highest correlation of any characteristic (chemical or physical) with DE and ME content (r = -0.42; *P* < 0.01; r = -0.43; *P* < 0.01), followed by ADF (r = -0.38; *P* < 0.01; r = -0.35; *P* < 0.01) and GE (r = 0.32; *P* < 0.05; r = 0.39; *P* < 0.05). Interestingly, in the 16 corn samples with a bulk weight between 662.0 and 765.9 g/L, bulk weight was not correlated with DE and ME content ( r = -0.39; *P* > 0.05; r = -0.26; *P* > 0.05) (Table 5).

**Table 5:** Correlation coefficients between chemical and physical characteristics and energy values of corn

**Item**	**DE**	**ME**	**Bulk weight**	**1,000 kernel weight**	**GE**	**Ash**	**EE**	**NDF**	**ADF**	**CP**
DE	1.00									
ME	0.95**	1.00								
Bulk weight	-0.39	-0.26	1.00							
1,000 kernel weight	0.12	0.07	0.21	1.00						
GE	0.32*	0.39*	0.17	-0.21	1.00					
Ash	0.32	0.27	-0.47	0.21	0.17	1.00				
EE	0.24	0.39	-0.05	-0.42	0.37	0.01	1.00			
NDF	-0.42*	-0.43*	0.27	-0.28	0.03	-0.09	-0.11	1.00		
ADF	-0.38*	-0.35*	0.20	-0.08	-0.02	0.10	-0.33	0.80**	1.00	
CP	0.21	0.04	-0.22	0.45	-0.26	0.21	-0.70	-0.54	-0.28	1.00

*, **, *P* < 0.05, *P* < 0.01, respectively.

## Discussion

### Relationships of physical characteristics and energy content

LS4 had the highest test weight and 1,000 kernel weight among the four varieties, but the results indicate that there is no significant correlation between physical characteristics (1,000 kernel weight and bulk weight) and available energy value in these grains. In the past, several attempts have been made to link the nutritional value of cereal grains to physical characteristics, such as density and kernel weight. Many researchers [12-14] concluded that available energy in growing pigs was not related to density in wheat samples. Likewise, in poultry, there were no correlations between ME and bushel weight or weight per 1,000 kernels in wheat samples [15,16]. In China, bulk weight is the most important marker of grade index for most feed companies. However, physical parameters such as bulk weight and 1,000 kernel weight cannot be used to estimate nutritional value of corn accurately for pigs.

The particle size of corn samples decreased with drying temperature, except for LS1. Drying temperature has substantial effects on breakage susceptibility, stress cracking, and dry-milling quality [17]. Increasing drying temperature increases breakage susceptibility [18,19]. The reason why particle size of LS1 was not affected by drying temperature may be that the LS1 was the soft corn hybrid.

### Effects of variety on the nutritive value of corn

Although special varieties of corn, such as high-oil, high-lysine and NutriDense, were not used in this experiment, the results of this study indicate that corn genetics significantly influence the 1,000 kernel weight and available energy content. The DE and ME are the most important parameters used in characterizing the nutritional value of cereal grains for livestock. Although the variety of LS1 was not a NutriDense corn, the DE content in LS1 was 2% greater than in LS4 corn; this is very valuable trait for feed companies. The reason for this observation was the ATTD of nutrients in the LS1 were greater than the other. It is likely that natural differences among varieties of corn exist, as has been demonstrated for corn and other cereal grains [14,20,21] and for sweet lupins [22].

### Effects of drying method and drying temperatures on the nutritive value of corn

The EE, NDF, ADF, CP, and ash contents were not significantly affected by drying temperature. The results are in agreement with those reported by Costa et al. [23]. A brown discoloration and a parched corn odor were also observed by Coates et al. [22] in corn dried at temperatures of 138 and 160°C. These characteristics suggest that a Maillard reaction has occurred in the grain kernel [24]. The parched odor of the corns dried at 120°C might have originated in similar fashion as the flavors from Maillard reaction described by Hathaway et al. [25] and Coates et al. [23]. In this study, drying method affected the bulk weight and 1,000 kernel weight. Other researchers have found that bulk weight and 1,000 kernel weight of corn generally decreases with increasing drying temperatures [16,19,26]. A factor that may affect bulk weight and 1,000 kernel weight is artificial drying, which removes moisture within the kernel without changing the kernel size.

With the exception of ATTD of EE, the available energy content (DE of DM; ME of DM) and ATTD of nutrients were not significantly affected by drying method. These results are in agreement with those reported by Costa et al. [23]. However, the ATTD of NDF and ADF for sundried corn samples were not significantly higher than for artificially dried samples. The ATTD of nutrients and available energy contents were not significantly different among corn dried at different temperatures. The percent of starch gelatinization did not increase with drying temperature. Our findings are not in agreement with increases in starch gelatinization percentage reported by Costa et al. [16]. The reason may be that the drying time, moisture, and processing condition in this study were not suited for starch gelatinization. Wood [26] showed that a high moisture content (30-50%) is required for efficient gelatinization. Furthermore, when limited amounts of water are present, more heating time is needed to complete drying [27]. Chiang and Johnson [28] and Della Valle et al. [29] reported that decreasing the retention time of the sample during processing decreased starch gelatinization. There was no extrusion process used in the present study, and the heating time was shorter than that of Costa et al. [16].

## Conclusions

In conclusion, variety had a significant impact on the nutritive value of corn for growing pigs. Heat treatment had some effects on the physical characteristics of corn, but this did not affect the nutritive value of corn for swine. These data suggest that greater accountability of these factors is required when assessing the nutritive value of corn for use in pig feed.

## Abbreviations

DE: Digestible energy; ME: Metabolizable energy; CP: Crude protein; NDF: Neutral detergent fibre; ADF: Acid detergent fibre; ATTD: Apparent total tract digestibility; EE: Ether extract; GE: Gross energy; DM: Dry matter; RSD: Residual standard deviation.

## Competing interests

The authors declare that they have no competing interests.

## Authors’ contributions

QFL carried out the experiment trial, performed the statistics and drafted the manuscript. SM and XSP participated in design of the study. DWL and SH participated animal trial. DFL and CHL conceived the study, and participated in its design and coordination. All authors read and approved the final manuscript.
